# Low-temperature redetermination of 1,3-bis­(penta­fluoro­phen­yl)triazene

**DOI:** 10.1107/S1600536810045447

**Published:** 2010-11-13

**Authors:** Karel G. von Eschwege, Annemarie Kuhn

**Affiliations:** a Department of Chemistry, University of the Free State, PO Box 339, Bloemfontein 9300, South Africa

## Abstract

The crystal structure of the title compound, (C_6_F_5_)_2_N_3_H, is stabilized by N—H⋯N hydrogen bonding, forming centrosymmetric dimers organized in a herringbone motif. Important geometrical parameters are N—N = 1.272 (2) and 1.330 (2) Å and N—N—N = 112.56 (15)°. The dihedral angle between C_6_F_5_ groups is 21.22 (9)°. The room temperature structure was reported by Leman *et al.* (1993). *Inorg. Chem.* **32**, 4324–4336]. In the current determination, the data were collected to a higher θ angle, resulting in higher precision for the C—C bond lengths(0.001–0.005 *versus* 0.003 Å).

## Related literature

Average bond lengths were obtained from the Cambridge Structural Database (Allen, 2000 ▶); For the synthesis of nitro­formazans, see: Pelkis *et al.* (1957 ▶); and for the synthesis of triazenes, see: Brooke *et al.* (1965 ▶). For use of triazenes in both synthesis and as metal coordinating ligands, see: Leman *et al.* (1993 ▶).
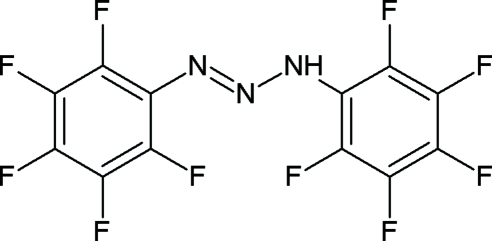


## Experimental

### 

#### Crystal data

C_12_HF_10_N_3_
*M* *_r_* = 377.16
Monoclinic, 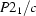

*a* = 9.9930 (8) Å
*b* = 9.4850 (8) Å
*c* = 12.9200 (11) Å
β = 95.585 (2)°
*V* = 1218.79 (18) Å^3^
*Z* = 4
Mo *K*α radiation
μ = 0.23 mm^−1^
*T* = 100 K
0.3 × 0.19 × 0.15 mm

#### Data collection

Bruker X8 APEXII 4K Kappa CCD diffractometer
Absorption correction: multi-scan (*SADABS*; Bruker; 2004 ▶) *T*_min_ = 0.933, *T*_max_ = 0.966
12525 measured reflections
3013 independent reflections
2177 reflections with *I* > 2σ(*I*)
*R*_int_ = 0.039

#### Refinement

*R*[*F* ^2^ > 2σ(*F* ^2^)] = 0.039
*wR*(*F* ^2^) = 0.107
*S* = 1.07
3013 reflections
226 parameters
H-atom parameters constrained
Δρ_max_ = 0.36 e Å^−3^
Δρ_min_ = −0.31 e Å^−3^

### 

Data collection: *APEX2* (Bruker, 2005 ▶); cell refinement: *SAINT-Plus* (Bruker, 2004 ▶); data reduction: *SAINT-Plus* and *XPREP* (Bruker, 2004 ▶); program(s) used to solve structure: *SIR97* (Altomare *et al.*, 1999 ▶); program(s) used to refine structure: *SHELXL97* (Sheldrick, 2008 ▶); molecular graphics: *DIAMOND* (Brandenburg & Putz, 2005 ▶); software used to prepare material for publication: *WinGX* (Farrugia, 1999 ▶).

## Supplementary Material

Crystal structure: contains datablocks global, I. DOI: 10.1107/S1600536810045447/kp2288sup1.cif

Structure factors: contains datablocks I. DOI: 10.1107/S1600536810045447/kp2288Isup2.hkl

Additional supplementary materials:  crystallographic information; 3D view; checkCIF report

## Figures and Tables

**Table 1:** Hydrogen-bond geometry (Å, °)

*D*—H⋯*A*	*D*—H	H⋯*A*	*D*⋯*A*	*D*—H⋯*A*
N3—H3⋯N1^i^	0.88	2.23	3.076 (2)	162

Symmetry code: (i) 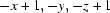.

**Fig. 1.**
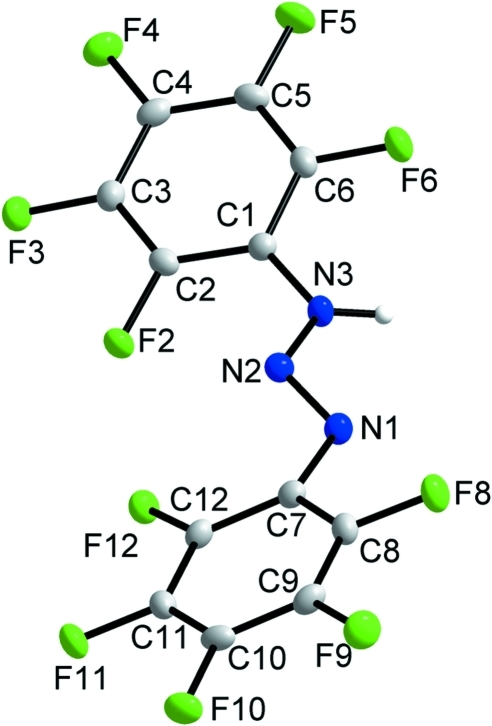
A view of (I). Displacement ellipsoids are drawn at the 30% probability level.

**Fig. 2.**
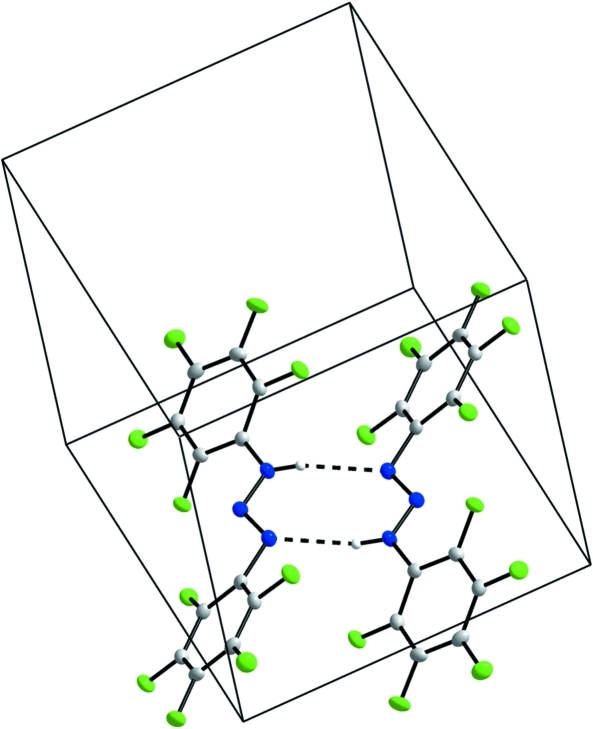
Hydrogen bonding of (I) showing the dimeric units formed.

**Fig. 3.**
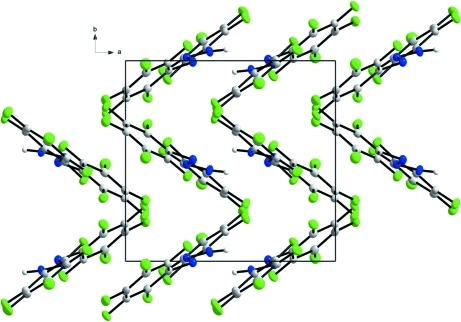
Packing diagram of (I) showing the herring-bone motif.

## supplementary crystallographic information

### Comment

A series of dithizones with varying number of fluorines on the phenyl rings were synthesised. During this process the nitroformazan precursor is initially prepared from the substituted aniline, as described by Pelkis *et al.*, 1957. In the final step the nitro group is substituted by sulfur, to form dithizone. However, during the first step to prepare pentafluorophenyldithizone it was found that, contrary to expectation, a triazene formed instead of the usual nitroformazan.  Previous studies show the extensive use of triazenes in both synthesis and as metal coordinating ligand, as illustrated by Leman *et al.*, 1993. Triazenes, in general, are traditionally synthesised slightly different, according to a method by Brooke *et al.*, 1965.

The title compound (Fig.1) and geometrical data are within expected values (average values obtained from the Cambridge Structural Database; Allen, 200). The C_6_F_5_ groups are twisted out of the plane formed by the N—N═N fragment 30.91 (16)° for ring C1—C6 and 38.49 (13)° for ring C7—C12. Hydrogen bonding (Fig. 2, Table 1) creates a centrosymmetric dimers that organise in a herring-bone motif along the *c* axis (Fig. 3).

### Experimental

Reagent chemicals and solvents were purchased from Sigma-Aldrich and used without further purification. For the synthesis 2,3,4,5,6 pentafluoroaniline (5 g, 27.3 mmol) was added to a mixture of concentrated hydrochloric acid (20 mL) and water (35 mL) at 273 K, and diazotized by the slow addition of sodium nitrite (3 g, 43 mmol), stirring for 30 min. The resulting solution was added, with stirring, to a cold mixture of sodium acetate (80 g), glacial acetic acid (45 mL) and water (25 mL). Nitromethane (5.0 g, 82 mmol) was added after 10 min. While heating at 343 K and stirring for 20 h the colour changed to dark-red. Filtering the precipitated 1,3 bis-pentafluorophenyltriazene off, gave the product in 52% yield. Crystals suitable for X-ray crystallography were grown from a mixture of dichloromethane and methanol. Although this method is typically used for the preparation of nitroformazan as precursor for the synthesis of dithizone, nitromethane did not serve here as coupling reagent in the synthesis of this highly fluorinated compound. The unexpected outcome is ascribed to the large electron-withdrawing capacity of the five fluorines on each phenyl ring.

### Refinement

The imine H atom was placed in a geometrically idealized position (C—H = 0.88) and constrained to ride on the parent atom with *U*_iso_(H) = 1.2*U*_eq_(C). The highest residual electron density peak of 0.36 e.Å is 0.70 Å from C9 and the deepest hole of -0.31 e.Å is 0.37 Å from H3 representing no physical meaning.

### Crystal data

**Table:**

C_12_HF_10_N_3_	*F*(000) = 736
*M**_r_* = 377.16	*D*_x_ = 2.055 Mg m^−^^3^
Monoclinic, *P*2_1_/*c*	Mo *K*α radiation, λ = 0.71073 Å
Hall symbol: -P 2ybc	Cell parameters from 2521 reflections
*a* = 9.9930 (8) Å	θ = 3.5–28.1°
*b* = 9.4850 (8) Å	µ = 0.23 mm^−^^1^
*c* = 12.9200 (11) Å	*T* = 100 K
β = 95.585 (2)°	Cuboid, red
*V* = 1218.79 (18) Å^3^	0.3 × 0.19 × 0.15 mm
*Z* = 4	

### Data collection

**Table:**

Bruker X8 APEXII 4K Kappa CCD diffractometer	3013 independent reflections
graphite	2177 reflections with *I* > 2σ(*I*)
Detector resolution: 8.4 pixels mm^-1^	*R*_int_ = 0.039
φ scans	θ_max_ = 28.3°, θ_min_ = 2.7°
Absorption correction: multi-scan (*SADABS*; Bruker; 2004)	*h* = −12→13
*T*_min_ = 0.933, *T*_max_ = 0.966	*k* = −12→12
12525 measured reflections	*l* = −17→17

### Refinement

**Table:**

Refinement on *F*^2^	Primary atom site location: structure-invariant direct methods
Least-squares matrix: full	Secondary atom site location: difference Fourier map
*R*[*F*^2^ > 2σ(*F*^2^)] = 0.039	Hydrogen site location: inferred from neighbouring sites
*wR*(*F*^2^) = 0.107	H-atom parameters constrained
*S* = 1.07	*w* = 1/[σ^2^(*F*_o_^2^) + (0.0438*P*)^2^ + 0.6444*P*] where *P* = (*F*_o_^2^ + 2*F*_c_^2^)/3
3013 reflections	(Δ/σ)_max_ < 0.001
226 parameters	Δρ_max_ = 0.36 e Å^−^^3^
0 restraints	Δρ_min_ = −0.31 e Å^−^^3^

### Special details

**Table:**

Experimental. The intensity data was collected on a Bruker X8 Apex II 4 K Kappa CCD diffractometer using an exposure time of 10 s/frame. A total of 1126 frames were collected with a frame width of 0.5° covering up to θ = 28.33° with 98.8% completeness accomplished.
Geometry. All e.s.d.'s (except the e.s.d. in the dihedral angle between two l.s. planes) are estimated using the full covariance matrix. The cell e.s.d.'s are taken into account individually in the estimation of e.s.d.'s in distances, angles and torsion angles; correlations between e.s.d.'s in cell parameters are only used when they are defined by crystal symmetry. An approximate (isotropic) treatment of cell e.s.d.'s is used for estimating e.s.d.'s involving l.s. planes.
Refinement. Refinement of *F*^2^ against ALL reflections. The weighted *R*-factor *wR* and goodness of fit *S* are based on *F*^2^, conventional *R*-factors *R* are based on *F*, with *F* set to zero for negative *F*^2^. The threshold expression of *F*^2^ > σ(*F*^2^) is used only for calculating *R*-factors(gt) *etc*. and is not relevant to the choice of reflections for refinement. *R*-factors based on *F*^2^ are statistically about twice as large as those based on *F*, and *R*- factors based on ALL data will be even larger.

### Fractional atomic coordinates and isotropic or equivalent isotropic displacement parameters (Å^2^)

**Table:**

	*x*	*y*	*z*	*U*_iso_*/*U*_eq_	
N1	0.32054 (15)	−0.00606 (18)	0.51342 (13)	0.0176 (4)	
N2	0.29181 (15)	0.01759 (18)	0.41693 (13)	0.0170 (4)	
N3	0.39984 (15)	0.04639 (18)	0.36826 (13)	0.0187 (4)	
H3	0.4793	0.0554	0.4034	0.022*	
C1	0.38491 (18)	0.0620 (2)	0.26046 (15)	0.0161 (4)	
C2	0.28659 (18)	−0.0055 (2)	0.19428 (16)	0.0170 (4)	
C3	0.27844 (18)	0.0129 (2)	0.08793 (16)	0.0183 (4)	
C4	0.37001 (19)	0.0977 (2)	0.04420 (16)	0.0198 (4)	
C5	0.46978 (19)	0.1626 (2)	0.10765 (16)	0.0199 (4)	
C6	0.47531 (18)	0.1467 (2)	0.21357 (16)	0.0184 (4)	
C7	0.20622 (17)	−0.0486 (2)	0.56306 (15)	0.0156 (4)	
C8	0.20040 (18)	−0.0106 (2)	0.66629 (16)	0.0178 (4)	
C9	0.09951 (19)	−0.0571 (2)	0.72416 (15)	0.0190 (4)	
C10	0.00216 (18)	−0.1471 (2)	0.67859 (16)	0.0187 (4)	
C11	0.00446 (18)	−0.1863 (2)	0.57591 (16)	0.0174 (4)	
C12	0.10529 (18)	−0.1381 (2)	0.51888 (15)	0.0165 (4)	
F2	0.19834 (11)	−0.09374 (13)	0.23200 (9)	0.0218 (3)	
F3	0.18284 (12)	−0.05460 (13)	0.02754 (9)	0.0254 (3)	
F4	0.36352 (12)	0.11346 (14)	−0.05941 (9)	0.0272 (3)	
F5	0.56138 (12)	0.24230 (14)	0.06517 (10)	0.0283 (3)	
F6	0.57237 (11)	0.21376 (13)	0.27471 (9)	0.0246 (3)	
F8	0.29674 (11)	0.07433 (13)	0.71246 (9)	0.0241 (3)	
F9	0.09641 (12)	−0.01701 (14)	0.82274 (9)	0.0269 (3)	
F10	−0.09396 (11)	−0.19674 (13)	0.73422 (10)	0.0243 (3)	
F11	−0.09111 (11)	−0.27180 (13)	0.53101 (10)	0.0245 (3)	
F12	0.10472 (11)	−0.18344 (13)	0.42082 (9)	0.0218 (3)	

### Atomic displacement parameters (Å^2^)

**Table:**

	*U*^11^	*U*^22^	*U*^33^	*U*^12^	*U*^13^	*U*^23^
N1	0.0153 (7)	0.0214 (9)	0.0163 (9)	0.0000 (6)	0.0024 (6)	−0.0012 (7)
N2	0.0160 (7)	0.0181 (9)	0.0172 (9)	0.0000 (6)	0.0031 (6)	−0.0005 (7)
N3	0.0142 (7)	0.0247 (10)	0.0172 (9)	−0.0037 (7)	0.0009 (6)	0.0012 (7)
C1	0.0152 (8)	0.0164 (10)	0.0167 (10)	0.0014 (7)	0.0017 (7)	0.0008 (8)
C2	0.0158 (8)	0.0147 (10)	0.0209 (11)	0.0001 (7)	0.0035 (7)	0.0003 (8)
C3	0.0173 (9)	0.0182 (10)	0.0190 (11)	0.0016 (8)	−0.0006 (8)	−0.0006 (8)
C4	0.0244 (10)	0.0200 (11)	0.0155 (10)	0.0055 (8)	0.0040 (8)	0.0026 (8)
C5	0.0193 (9)	0.0177 (11)	0.0236 (11)	0.0004 (8)	0.0070 (8)	0.0035 (9)
C6	0.0150 (8)	0.0167 (10)	0.0230 (11)	−0.0002 (7)	−0.0003 (8)	−0.0013 (8)
C7	0.0138 (8)	0.0171 (10)	0.0159 (10)	0.0027 (7)	0.0014 (7)	0.0014 (8)
C8	0.0144 (8)	0.0186 (11)	0.0199 (11)	0.0000 (7)	−0.0003 (7)	−0.0018 (8)
C9	0.0210 (9)	0.0226 (11)	0.0134 (10)	0.0053 (8)	0.0023 (8)	0.0014 (8)
C10	0.0163 (9)	0.0192 (11)	0.0215 (11)	0.0035 (8)	0.0060 (8)	0.0069 (8)
C11	0.0155 (8)	0.0152 (10)	0.0211 (11)	−0.0005 (7)	−0.0009 (7)	0.0004 (8)
C12	0.0176 (9)	0.0187 (10)	0.0130 (10)	0.0025 (7)	0.0004 (7)	−0.0002 (8)
F2	0.0208 (5)	0.0244 (7)	0.0203 (6)	−0.0077 (5)	0.0023 (5)	0.0023 (5)
F3	0.0293 (6)	0.0275 (7)	0.0183 (7)	−0.0069 (5)	−0.0036 (5)	−0.0011 (5)
F4	0.0334 (7)	0.0318 (8)	0.0171 (7)	0.0016 (6)	0.0055 (5)	0.0032 (5)
F5	0.0265 (6)	0.0308 (7)	0.0292 (7)	−0.0082 (5)	0.0106 (5)	0.0064 (6)
F6	0.0197 (5)	0.0285 (7)	0.0252 (7)	−0.0087 (5)	−0.0005 (5)	−0.0005 (5)
F8	0.0226 (6)	0.0298 (7)	0.0195 (7)	−0.0071 (5)	0.0004 (5)	−0.0053 (5)
F9	0.0304 (6)	0.0342 (8)	0.0168 (6)	−0.0011 (5)	0.0060 (5)	−0.0033 (5)
F10	0.0212 (6)	0.0270 (7)	0.0260 (7)	−0.0008 (5)	0.0098 (5)	0.0048 (5)
F11	0.0198 (5)	0.0260 (7)	0.0274 (7)	−0.0088 (5)	−0.0002 (5)	−0.0008 (5)
F12	0.0241 (6)	0.0249 (7)	0.0162 (6)	−0.0046 (5)	0.0018 (5)	−0.0029 (5)

### Geometric parameters (Å, °)

**Table:**

N1—N2	1.272 (2)	C5—C6	1.373 (3)
N1—C7	1.422 (2)	C6—F6	1.349 (2)
N2—N3	1.330 (2)	C7—C8	1.388 (3)
N3—C1	1.394 (2)	C7—C12	1.397 (3)
N3—H3	0.88	C8—F8	1.349 (2)
C1—C6	1.391 (3)	C8—C9	1.385 (3)
C1—C2	1.394 (3)	C9—F9	1.332 (2)
C2—F2	1.341 (2)	C9—C10	1.382 (3)
C2—C3	1.380 (3)	C10—F10	1.340 (2)
C3—F3	1.336 (2)	C10—C11	1.380 (3)
C3—C4	1.380 (3)	C11—F11	1.341 (2)
C4—F4	1.342 (2)	C11—C12	1.383 (3)
C4—C5	1.373 (3)	C12—F12	1.337 (2)
C5—F5	1.345 (2)		
			
N2—N1—C7	112.19 (15)	F6—C6—C1	118.53 (18)
N1—N2—N3	112.56 (15)	C5—C6—C1	122.21 (18)
N2—N3—C1	118.78 (15)	C8—C7—C12	117.02 (17)
N2—N3—H3	120.6	C8—C7—N1	118.04 (17)
C1—N3—H3	120.6	C12—C7—N1	124.61 (18)
C6—C1—C2	116.55 (18)	F8—C8—C9	118.51 (18)
C6—C1—N3	119.05 (17)	F8—C8—C7	119.02 (17)
C2—C1—N3	124.38 (17)	C9—C8—C7	122.46 (18)
F2—C2—C3	117.62 (17)	F9—C9—C10	120.19 (17)
F2—C2—C1	120.85 (18)	F9—C9—C8	120.73 (18)
C3—C2—C1	121.51 (18)	C10—C9—C8	119.08 (18)
F3—C3—C2	119.36 (17)	F10—C10—C11	120.04 (18)
F3—C3—C4	120.34 (18)	F10—C10—C9	120.00 (18)
C2—C3—C4	120.28 (18)	C11—C10—C9	119.95 (17)
F4—C4—C5	120.47 (18)	F11—C11—C10	120.08 (17)
F4—C4—C3	120.24 (18)	F11—C11—C12	119.65 (18)
C5—C4—C3	119.27 (19)	C10—C11—C12	120.28 (18)
F5—C5—C6	120.39 (18)	F12—C12—C11	117.55 (17)
F5—C5—C4	119.46 (19)	F12—C12—C7	121.24 (17)
C6—C5—C4	120.14 (18)	C11—C12—C7	121.18 (18)
F6—C6—C5	119.25 (17)		
			
C7—N1—N2—N3	−174.92 (16)	N3—C1—C6—C5	177.51 (18)
N1—N2—N3—C1	174.28 (17)	N2—N1—C7—C8	−147.83 (18)
N2—N3—C1—C6	152.97 (18)	N2—N1—C7—C12	38.9 (3)
N2—N3—C1—C2	−29.0 (3)	C12—C7—C8—F8	179.22 (17)
C6—C1—C2—F2	177.31 (17)	N1—C7—C8—F8	5.4 (3)
N3—C1—C2—F2	−0.7 (3)	C12—C7—C8—C9	−0.5 (3)
C6—C1—C2—C3	−1.0 (3)	N1—C7—C8—C9	−174.24 (18)
N3—C1—C2—C3	−179.05 (18)	F8—C8—C9—F9	1.1 (3)
F2—C2—C3—F3	1.3 (3)	C7—C8—C9—F9	−179.21 (18)
C1—C2—C3—F3	179.65 (17)	F8—C8—C9—C10	−178.35 (17)
F2—C2—C3—C4	−177.26 (17)	C7—C8—C9—C10	1.3 (3)
C1—C2—C3—C4	1.1 (3)	F9—C9—C10—F10	−1.5 (3)
F3—C3—C4—F4	0.2 (3)	C8—C9—C10—F10	177.96 (17)
C2—C3—C4—F4	178.76 (17)	F9—C9—C10—C11	178.85 (17)
F3—C3—C4—C5	−178.06 (18)	C8—C9—C10—C11	−1.7 (3)
C2—C3—C4—C5	0.5 (3)	F10—C10—C11—F11	1.4 (3)
F4—C4—C5—F5	−0.1 (3)	C9—C10—C11—F11	−178.93 (17)
C3—C4—C5—F5	178.22 (18)	F10—C10—C11—C12	−178.43 (17)
F4—C4—C5—C6	179.63 (18)	C9—C10—C11—C12	1.2 (3)
C3—C4—C5—C6	−2.1 (3)	F11—C11—C12—F12	−2.0 (3)
F5—C5—C6—F6	1.1 (3)	C10—C11—C12—F12	177.86 (17)
C4—C5—C6—F6	−178.58 (18)	F11—C11—C12—C7	179.80 (17)
F5—C5—C6—C1	−178.10 (18)	C10—C11—C12—C7	−0.3 (3)
C4—C5—C6—C1	2.2 (3)	C8—C7—C12—F12	−178.17 (17)
C2—C1—C6—F6	−179.85 (17)	N1—C7—C12—F12	−4.9 (3)
N3—C1—C6—F6	−1.7 (3)	C8—C7—C12—C11	0.0 (3)
C2—C1—C6—C5	−0.6 (3)	N1—C7—C12—C11	173.28 (18)

### Hydrogen-bond geometry (Å, °)

**Table:**

*D*—H···*A*	*D*—H	H···*A*	*D*···*A*	*D*—H···*A*
N3—H3···N1^i^	0.88	2.23	3.076 (2)	162

Symmetry codes: (i) −*x*+1, −*y*, −*z*+1.
